# Contemporary Hearing Rehabilitation Options in Patients with Aural Atresia

**DOI:** 10.1155/2014/761579

**Published:** 2014-04-29

**Authors:** Jacky F. W. Lo, Willis S. S. Tsang, Joannie Y. K. Yu, Osan Y. M. Ho, Peter K. M. Ku, Michael C. F. Tong

**Affiliations:** ^1^Department of ENT, United Christian Hospital, Kwun Tong, Hong Kong; ^2^Department of ENT, Prince of Wales Hospital, Shatin, Hong Kong; ^3^Department of Otorhinolaryngology, Head and Neck Surgery, The Chinese University of Hong Kong, Hong Kong

## Abstract

Congenital aural atresia is the failure of development of the external auditory canal. It usually occurs in conjunction with microtia, which is the malformation of the auricle due to a failure of development of the external ear. Aural atresia, with or without microtia, may significantly affect the hearing and social life of the patients. It is important for every medical practitioner to be aware of the possible treatment options for hearing rehabilitation in this group of patients. In the era of modern technology, new choices, including Bone-Anchored Hearing Aid (BAHA) (Cochlear Ltd. and Oticon Medical), Vibrant Soundbridge (VSB) (MED-EL, Innsbruck, Austria), and Bonebridge system (BB) (MED-EL, Innsbruck, Austria), provide high-end alternatives to traditional Bone Conduction Hearing Aid and Auditory Canal Reconstruction. All these options have advantages and disadvantages, and they are appropriate for different patients and/or at different ages. This paper aims to provide an overview of the management of hearing rehabilitation in congenital aural atresia patients and a discussion of each treatment option.

## 1. Overview of Aural Atresia

Aural Atresia is estimated to have an incidence of 1 : 10,000 to 1 : 20,000 [1, 2]. The majority of the cases are unilateral and male-predominant, 2.5 times more prevalent than female. It is often described that cases on the right side are more common. Most cases of atresia are associated with microtia and the degree of atresia is correlated with the degree of auricular deformity [1]. The inner ear is less associated with concurrent deformity due to the difference in embryological origin. This phenomenon also forms a basis for hearing rehabilitation in aural atresia patients.

Most of the cases are isolated and sporadic; sometimes it is associated with syndromes like Treacher Collins, Goldenhar, and Pierre Robin syndrome or chromosome abnormalities like deletions of long arm of chromosome 18 [2, 3].

## 2. Management

Patients with atresia and microtia should have both problems tackled. Through years of refinement, microtia surgery can now provide a high-quality autologous reconstruction of the external ear, but this will not be covered here. In the following paragraphs, the focus will be put on hearing rehabilitation of atresia patients.

The majority of the patients (80–90%) have moderate severe to severe grade conductive hearing loss on the diseased side [4]. Patients with* unilateral* aural atresia usually have normal hearing on the unaffected side, unless an underlying syndrome is associated, for example, Goldenhar syndrome [5]. It is essential to identify those with* bilateral hearing loss* early as they need early intervention to provide adequate stimulation for speech and language development.

Despite intact hearing on the contralateral side, it has been well established that patients with unilateral hearing loss have significant difficulties in academic performance and communication [6, 7]. They also suffer from lower self-esteem and at least 25% of the patients' parents and teachers report behavioural problems and academic performance issues [8]. It is even reported that in severe and profound unilateral loss, the Intelligence Quotient (IQ) may be significantly lower than normal [9]. In daily living, they often complain of poor stereotactic sense due to the problem of head shadowing. Not only is the hearing aspect affected, but also children with unilateral hearing loss may have poorer oral expression and oral composition [10].

## 3. Clinical Evaluation

Every patient with aural atresia should have an audiological evaluation to assess the type and the degree of hearing loss. They also need a computer tomogram to delineate the anatomy of the middle ear and inner ear, so as to decide the best option for the hearing rehabilitation.

Various classifications of congenital aural atresia exist, based on the clinical and/or radiological appearance. Probably the most clinically useful is the Jahrsdoerfer classification, described in 1992. It is a 1 to 10 point scoring system based on CT findings (Table 1). Each presence of normal anatomy scores 1 point and the presence of the stapes scores 2 points, signifying its importance. This scoring system helps decide whether atresia reconstruction surgery is likely to be successful for each patient [11].

## 4. Treatment Options

Modern technology has brought more treatment options for congenital aural atresia. Implantable hearing aids including BAHA, BB, and VSB offer more choices for our patients. It is essential to discuss thoroughly the different characteristics and pros and cons of each option with the patient and their family, before the final decision is made.

*Option  1: No Treatment*. This option is for those with* unilateral* aural atresia without speech and language developmental delay [12]. There was a controversy about how much we should do for patients with* unilateral* congenital aural atresia, as they usually had normal hearing on the contralateral side. Therefore, some otologists hesitated to intervene early in paediatric patients [13]. But more and more studies have proved that restoring binaural hearing can bring more benefits than harm, including better hearing in noise, improved distance hearing and elimination of head-shadowing, and better sound localization [13]. However, It should be borne in mind that every patient's needs and expectations vary; a detailed discussion should be held to achieve the best solution for each individual case.

**Figure 1:**
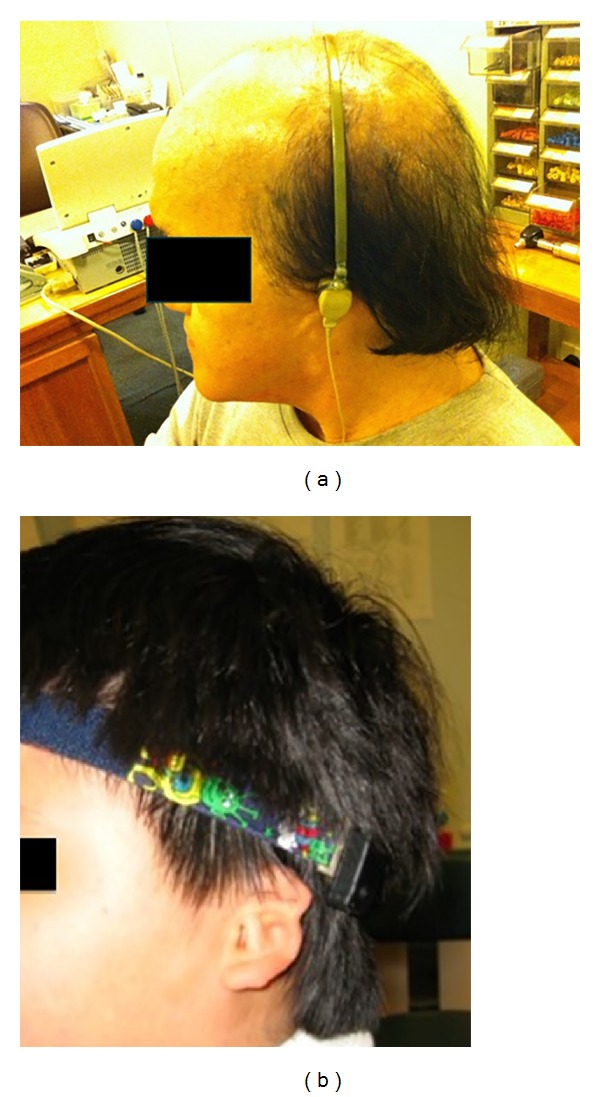
Bone conduction hearing aid. (a) Conventional headband, (b) softband BAHA.

*Option  2: Bone Conduction Hearing Aid*. The mechanism of the bone conduction hearing aid is simple. Sound is picked up by the microphone and is translated to the bony skull via a vibrator. The vibrator has to be pressed tightly on the mastoid to achieve good sound conduction. Despite its simplicity, this system is very visible and the firm pressure over the mastoid skin causes discomfort and skin problems. Moreover, it may hinder an active life-style or participation in sport [14]. In our centre, this option was used only for those unsuitable for surgical hearing rehabilitation. It is prescribed either as headband (Figure 1(a)) or softband BAHA fitting (Figure 1(b)).

*Option  3: Canalplasty*. Canalplasty or Atresiaplasty, the reconstruction of the external auditory canal, was first attempted by Kiesselbach in 1883 [3]. Patients suitable for this surgery must have a normally functioning cochlear, as demonstrated by CT and audiogram. Nowadays, candidates for canalplasty are selected according to the Jahrsdoerfer classification in our unit. Canalplasty is only attempted in the group of patients with score of 5 or above [1]; some authors suggested that candidates with a Jahrsdoerfer scale score of 7 or greater are preferred to achieve a higher degree of audiometric outcome after the surgery [13].

The aim of canalplasty is to achieve a clean, dry, and patent external auditory canal and thus provide serviceable hearing. If it succeeds, it can provide the best opportunity for life-long, amplification-free hearing.

The mean postoperative speech reception threshold is 25–35 dB HL, which is the range of mild hearing loss and around 30% of patients still need to have a conventional hearing aid to assist with hearing after surgery [3].

In addition, otologists are often frustrated with the relatively common occurrence of restenosis and recurrent infections of the canal. The reported rate of restenosis ranges from 5 to 29% [3, 15, 16]. Around 26% of the population requires reoperation [3]. Serious complications may also happen in this surgery, such as worsening of hearing loss and facial nerve palsy in 1% of the cases [1], as up to 30% of this group of patients have an anomaly in the course of their facial nerve [17].

Another drawback of this surgery is that it requires the patient's cooperation and participation for postoperative toileting and dressing. The age and maturity of the patient are therefore also relevant factors in deciding whether this type of surgery is appropriate. In our unit, we normally carry out the surgery after 8–10 years of age.

*Option  4: Implantable Hearing Aids*. There are three types of implantable hearing aids; Bone-Anchored Hearing Aids (BAHA), Bonebridge (BB), and Vibrant Soundbridge (VSB).

### 4.1. Bone Anchored Hearing Aid (BAHA) 

#### 4.1.1. Background

**Figure 2:**
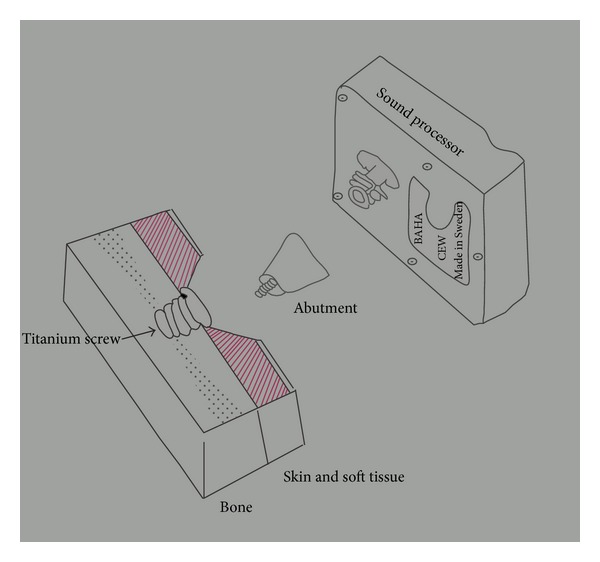
Schematic diagram of BAHA implantation.

BAHA (Figure 2) combines the concept of osseointegration and bone conduction transmission to aid hearing. Osseointegration refers to the direct structural and functional connection between ordered living bone and the surface of a load-carrying implant [18]. It was found in the 1950s by Professor Branemark that implanted titanium would fuse with human bone in harmony and become part of the bone, instead of giving rise to a foreign body reaction [14]. Tjellstron was the pioneer in utilizing the concept of osseointegration in hearing implants and established the use of BAHA in 1977 [18].

#### 4.1.2. Principle and Surgical Technique

**Figure 3:**
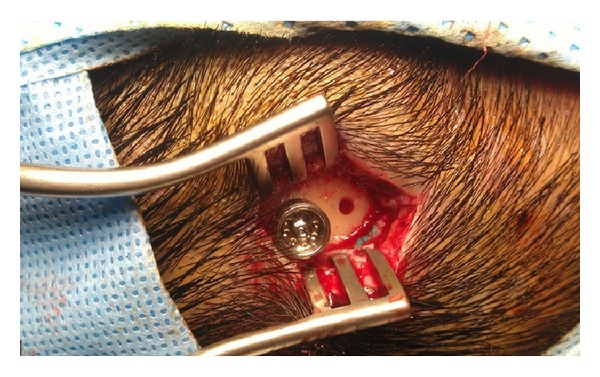
Intraoperative photo of BAHA surgery showing the installation of abutment.

BAHA is a percutaneous implantable hearing system, consisting of a titanium fixture, abutment, and a sound processor. The titanium fixture is implanted in the skull bone of the patient and attached to a percutaneous abutment and sound processor. The sound processor will convert sound energy to vibration, transmitted via the abutment and the titanium fixture and then the skull, directly to the functioning cochlea via bone conduction (Figure 3).

A small incision is all one needs. A 4 mm titanium fixture is secured to the bone. The abutment is then joined to the fixture after thinning the periabutment subcutaneous tissue. The sound processor is fitted 3 months after the fixture has fully osseointegrated with the skull bone.

**Figure 4:**
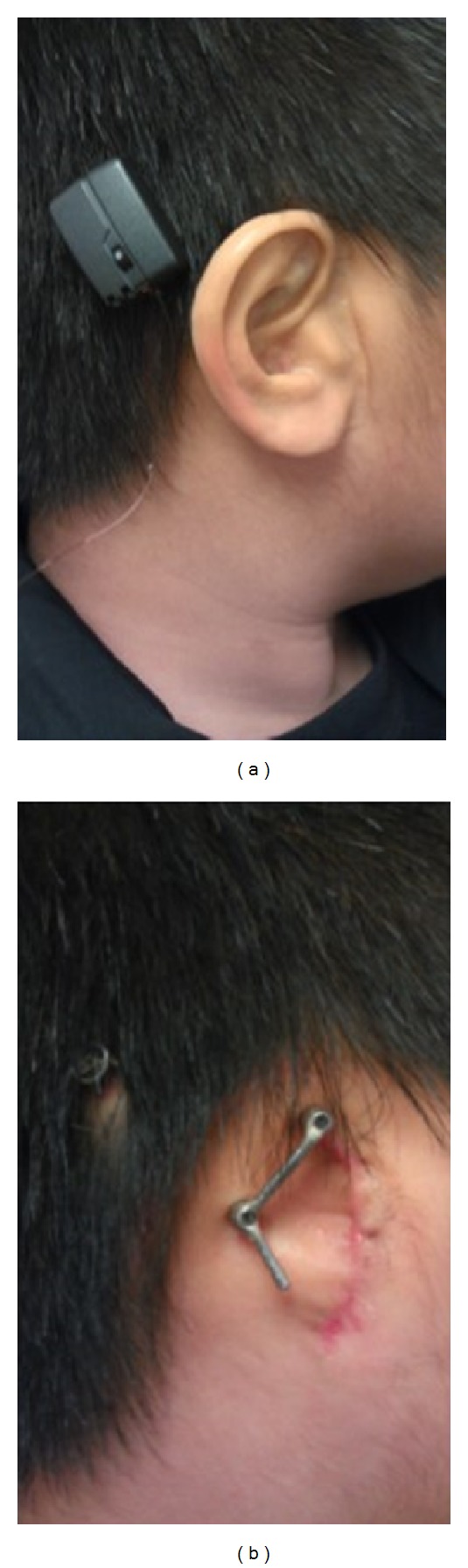
(a) A Child with Bone Anchored Hearing Aid. (b) This patient also has a prosthetic ear with osseointegrated implant.

A skull thickness of 3 to 4 mm and good bone quality are essential for successful BAHA surgery. In paediatric patients with a thinner skull, it is recommended that surgery is performed in two stages. The first stage is the insertion of the titanium fixture. Instead of proceeding further, the wound is closed to allow time for osseointegration, so as to secure the fixture. In the second stage, which is approximately 3 months later, the abutment is placed. The sound processor can be fitted 3 weeks after that (Figures 4(a) and 4(b)). Sometimes, a 3 mm titanium fixture is needed for patients with thinner skull bone. A two-stage procedure is also indicated for patients with a history of irradiation to the skull.

#### 4.1.3. Risks and Benefits

BAHA system implantation is a relatively simple and safe procedure. The most common complications are soft tissue problems and failure of osseointegration. Serious complications, such as dura tear and CSF leak are extremely rare.

Failure of osseointegration is more common in children especially in syndromal children with thinner skulls [18]. Cass and Mudd [18] reported a failure rate of 78% for the 3 mm fixture, while that of the 4 mm fixture is only 13%. Previous irradiation, age, and surgical technique also influence the chances of good osseointegration.

Soft tissue complications include infection, skin reactions, or granulation formation and overgrowth into the abutment. These are more common in children, as postoperative dressing and good hygiene are more difficult to maintain in children. For Mazita et al. [19], among their 16 patients, only one had failure of osseointegration requiring revision surgeries and the overall complication rate was about 20%; the complications were granulation and cellulitis. In our center, the BAHA complication rate is about 5%.

BAHA gains its reputation from its simplicity and effectiveness. Generally speaking, for those with bilateral conductive hearing loss, BAHA can bring a reduction of air-bone gap to 10 dB HL in 80% of patients and complete closure of the gap in 30% of patients [20]. In studies that specifically look into the use of BAHA in atresia patients, Mazita et al. [19] had found that 100% (16 patients) have improved hearing with a mean functional gain of 35.2 dB HL. In another study published by Ricci et al. [21], 31 patients received BAHA implantation for bilateral aural atresia, the mean postoperative free-field threshold of 18.1 +/− 7.5 dB HL, with ABG closure in 24 out of 31 patients (77.4%).

Apart from the improvement in hearing, quality of life (QOL) of patients implanted with BAHA was also improved, 30 out of 31 patients reported improvement in QOL with evaluation using Glasgow Benefit Inventory [21]. This feature is also supported by a large number of other studies [22, 23].

In a study recently published in the USA [24], comparison between BAHA implant and patient with EAC reconstruction was conducted. It is found that BAHA implantation has resulted in a significant hearing gain than the reconstruction group (44.3 versus 20.0 dB HL, *P* < 0.001) despite the fact that quality of life assessment showed no significant difference, and similar complication rates are noted in both groups.

For children with* unilateral *hearing loss or unilateral atresia, they are recommended to have BAHA surgery if the headband BAHA trial (Figure 1(b)) shows improvement in hearing in noise or sound localization.

### 4.2. Bonebridge (BB) 

#### 4.2.1. Background

**Figure 5:**
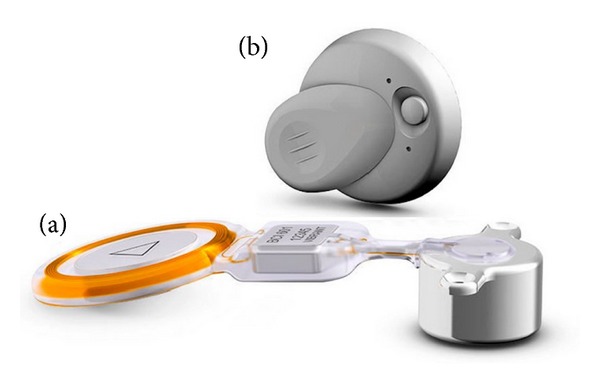
Bonebridge system. (a) The implant, (b) the audio processor.

Despite its simplicity, patients on BAHA need to have a lifelong commitment to wound care. Periabutment infection is a particular problem, especially in countries with a hot and humid weather, like Hong Kong. This may eventually require surgical retrieval of the implant. In response to this common problem, BB (Figure 5) was developed.

#### 4.2.2. Principle and Surgical Technique

BB is a device consisting of an audio processor (external) and a Bone Conduction Implant (BCI). Unlike BAHA, sound received by the processor is transmitted to the BCI transcutaneously via an electromagnetic field. The BCI consists of a receiver coil, a demodulator, and a transducer. The receiver receives the signal, routes it through the demodulator, and then the transducer converts sound energy into vibration. The sound signal is thus received by the cochlea by means of bone conduction. The use of transcutaneous electromagnetic transmission obviates the need for a physical pin tract, and thus it eliminates the possibility of wound complications.

**Figure 6:**
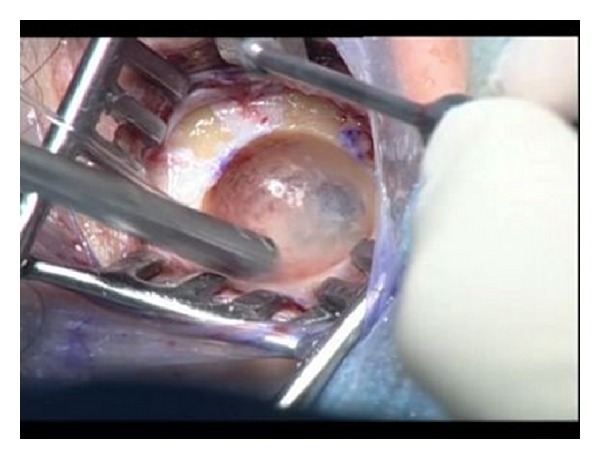
Bonebridge surgery: drilling on the mastoid bone to create a cavity for inserting floating mass transducer.

**Figure 7:**
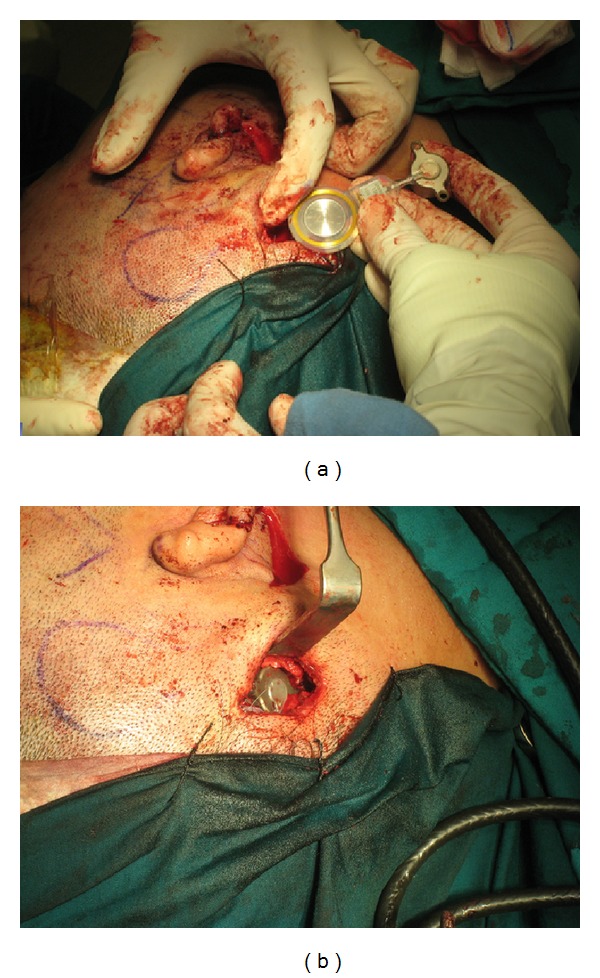
(a) A right microtia patient receiving Bonebridge implantation. (b) Right Bonebridge implant in place.

The transducer part of the BCI is sizable with a thickness of 8.7 mm; therefore, an area of the skull with thickness over 8.7 mm is needed. This area is carefully selected in the CT scan preoperatively, and is usually in the mastoid region. A postauricular approach is employed and a hole that fits the transducer is drilled (Figure 6). Once the BCI is fitted in and secured, the wound is closed (Figures 7(a) and 7(b)). The device can be activated two weeks later. This is much earlier than BAHA implantation as osseointegration is not required for the bone conduction signal transmission in Bonebridge device.

#### 4.2.3. Risks and Benefits

A certain thickness of skull bone is needed for the implantation of the transducer. This limits the eligibility of patients for this surgery, and only patients of 18 years old are suitable. BB surgery is not technically demanding and can even be considered simpler than a cortical mastoidectomy. The uncommon but serious complication is the injury to the sigmoid sinus and the dura, as drilling takes place directly over the sinodural angle [23].

BB implantation is a relatively new innovation and thus few studies discussed its clinical outcome. Manrique et al. [25] described a gain of 35.62 dB HL in PTA after Bonebridge implanted for patients with mixed hearing loss. Interestingly, 1000 Hz was the frequency with most significant improvement, whereas 250 Hz was found to have the least change in hearing threshold. It is said that this is a phenomenon observed typically in bone-anchored hearing aid. The word discrimination percentage, in his study, was also shown to have statistically significant improvement (20%). Another study from Barbara's group [26] agreed with Manrique et al. study that average PTA gain, in mixed hearing loss patients, is about 36.5 dB HL after Bonebridge was implanted and all patients have 100% speech recognition after the procedure.

**Figure 8:**
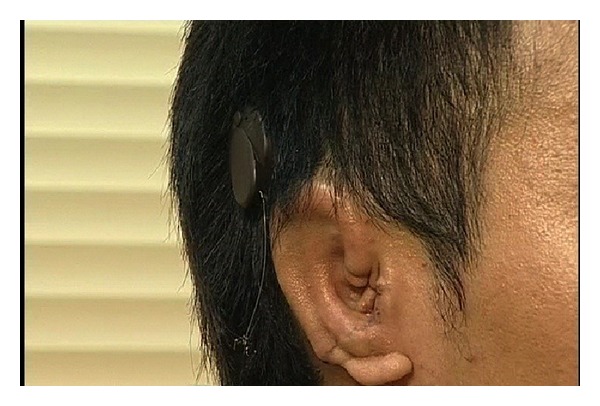
A patient wearing Bonebridge. He also had Stage 1 microtia reconstruction done.

In Hong Kong, we have over 15 patients that have implanted BB, with careful candidacy selections as well as detailed preoperative and postoperative managements, all patients were satisfied with the BB device and wearing it on regular basis (Figure 8).

### 4.3. Vibrant Soundbridge (VSB) 

#### 4.3.1. Background

**Figure 9:**
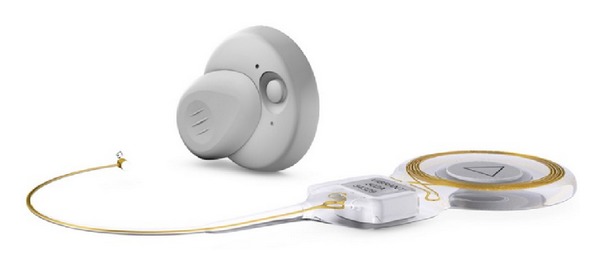
The Vibrant Soundbridge. The implant and the sound processor.

VSB (Figure 9) differs from BAHA and BB in that it provides stimulation to the inner ear via a different mechanism. BAHA and BB aid hearing by transmitting sound through bone conduction; hence, both inner ears will be stimulated. In some patients, this may result in signal confusion and thus incorrect location of sound direction. VSB, on the other hand, is implanted inside one middle ear, which provides unilateral stimulation to the inner ear system. This unilateral direct inner ear stimulation completely removes the possibility of signal confusion.

#### 4.3.2. Principle and Surgical Technique

VSB is a middle ear implant consisting of two parts, the External Audio Processor (AP) and the implantable Vibrating Ossicular Prosthesis (VORP). The AP picks up sound signals, amplifies them, and transmits them to the VORP. The Floating Mass Transducer (FMT), in the distal part of the VORP, vibrates the attached middle ear structure through a single point of attachment and thereby stimulates the cochlea.

**Figure 10:**
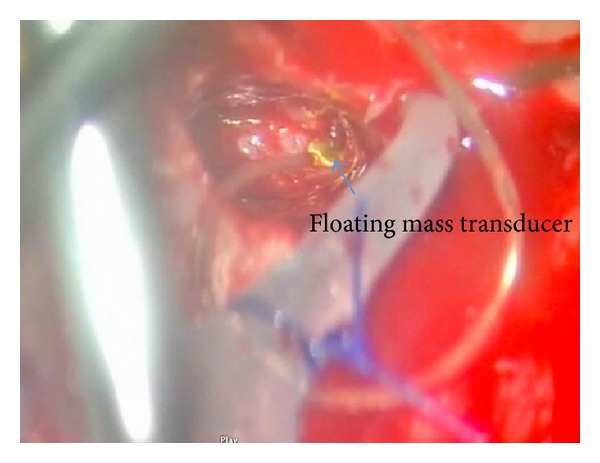
Right round window vibroplasty. The floating mass transducer sitting on round window niche.

**Figure 11:**
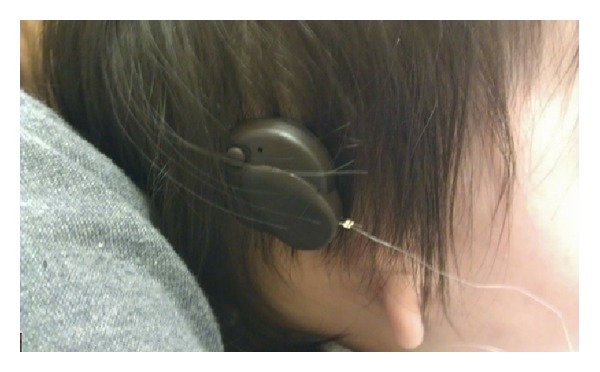
Postimplantation of Vibrant Soundbridge.

In aural atresia patients, the FMT can either be attached to the stapes, if it is functioning, or to the round window, in which case the procedure is known as round window vibroplasty (Figure 10). The device can be activated 8 weeks after the operation (Figure 11).

#### 4.3.3. Risks and Benefits

VSB implantation is a more sophisticated surgery. The usually malformed middle ear cleft has to be entered for inserting the FMT. It carries a risk of injury to facial nerves and inner ear, while no such complications would occur in BAHA or BB. Facial nerve monitoring is therefore essential during the surgery. Intraoperative audiological monitoring is also needed to make sure the position of the FMT is satisfactory.

The official recommendation for VSB is for patients older than 3 years. However, the Colletti group has been working on VSB in infants and younger children. Their results are positive for implantation in patients as young as 2 months old [27]. We advocate the installation of VSB in patients of 18 months old, by which time the middle ear can comfortably accommodate the FMT. The patient by this age should also be able to comply with the postoperative hearing and speech rehabilitation program.

The effectiveness and superiority of VSB has been proven by many studies. Frenzel group presented 7 patients with VSB surgery for unilateral atresia, and it was found that the mean hearing again was 45.5 dB HL and the speech reception threshold was 21 dB SPL, compared with pre-VSB 59 dB SPL. No perioperative complication was experienced. In an even earlier study in the USA in 2007 [4], 5 patients with aural atresia had VSB installed, and patients experienced an improvement of the sound-field thresholds up to 50 dB; an average improvement of 70% in speech discrimination at a sound level of 65 dB HL. The average speech threshold showed a functional gain of 32 dB HL.

In another new study from Argentina [28], an even higher functional gain is showed with 55.1 dB HL in 12 patients with osseous atresia.

From the experience of our centre [29], VSB can bring an average of 11 dB of audibility and also provide significant improvement in speech intelligibility, evaluated at 3 and 6 months after device activation.

The position of floating mass transducer which is to be put remained controversial. Some authors suggested the outcome was better with FMT placed over oval window and stapes crura [28], while the others suggested that there was no significant difference [30].

## 5. What to Choose?

With the development of new methods of implantation, the flexibility of hearing rehabilitation has greatly increased. However, it is never easy to make the final call on which method will be best. Factors like age, type of microtia and aural atresia, background medical history, and patient's expectations should be taken into account. The ultimate decision should always be an agreement among the patients, their parents, and the hearing rehabilitation team, after a thorough discussion.

The following provides our centre's approach in counseling patients with atresia.

### 5.1. Unilateral Atresia versus Bilateral Atresia

Patients with* bilateral *atresia should have either a softband BAHA or a conventional headband bone conduction hearing aid in early life to provide adequate stimulation for the development of the central nervous system. They should promptly be referred to the otology center. Then further long-term hearing rehabilitation should be considered when the patient grows older, as discussed below.

For those with* unilateral* disease without speech and language developmental delay, time is allowed for a thorough discussion and consideration of treatment options.

### 5.2. Age

The suitable ages for hearing aid implantation differ among each treatment option, as some of the devices require certain skull bone thickness. For patients less than 18 months old with microtia and atresia, the choices for hearing rehabilitation are softband BAHA and conventional headband bone conduction hearing aids. For those older than 18 months, they can opt for the VSB. When the child grows older, percutaneous BAHA becomes an option; usually by 5 years old. Canalplasty is a choice for selected candidates after the age of 8. Bonebridge was initially designed for patients over 18 years old because certain thickness of the cortical bone is required; recently, design of Bonebridge is modified and has been successfully implanted in paediatric patients.

### 5.3. Patient's Medical History

In some patients who need repeated Magnetic Resonance Imaging (MRI), for example in patients with neurological diseases, the method of hearing rehabilitation has to be carefully considered. This is because MRI is generally not recommended for patients with implantable hearing aids, like VSB. BB can tolerate MRI scanning up to 1.5 tesla and BAHA up to 3 tesla. These implants also produce artifacts in images of the brain. BAHA is the least disturbing due to its small implant size.

It should also be noted that monopolar diathermy could no longer be used in patients with BB or VSB.

### 5.4. Anatomy

The anatomy of the temporal bone is a major confounding factor in the approach of hearing rehabilitation. Patients with good middle ear anatomy (Jahrsdoerfer score 7 or above) may be suitable for canal reconstructions. However, the presence of dehiscent facial nerve or high riding jugular bulb may add substantial risks to VSB implantation. Also the skull bone thickness governs the possibility of BAHA or BB implantation.

### 5.5. Patient's Expectations

Hearing rehabilitation is not just a one-off procedure. Patient participation contributes the most to the success of the surgery. Canalplasty requires patient to have regular and frequent cleaning and wound dressing, at least in the early post-op period. BAHA requires patient to dress their wound daily and have a high quality of wound hygiene for life. Tuning of implantable devices can be a time-consuming procedure and it is vital to have the full engagement of both audiologist and patient during this postoperation period.

### 5.6. Hospital Rehabilitation Team

Multidisciplinary support is vital to success. In our unit, doctors, nursing staff, audiologists, speech therapists, and social workers work together to deliver a high quality comprehensive service to atresia patients. For example, after BAHA surgery, a team of nursing staff is dedicated to post-op wound management. They play an important role in minimizing the post-op infection rate. The nursing team also helps patients to deal with any difficulties in post-op care and mental stress. Audiologists support patients with device related problems. Speech therapist follows the patients to help their language development after the implantation.

In summary, most patients with aural atresia benefit from hearing rehabilitation. The choices are conventional headband bone conduction hearing aid, softband BAHA, canalplasty, percutaneous BAHA, VSB, and BB. Each option has its strengths and weaknesses. The newly developed BB and VSB devices have provided excellent hearing rehabilitation ability to those atresia patients and are gaining in popularity. Early identification and referral for further management are the key to obtain a successful long-term outcome.

## Figures and Tables

**Table 1:** Jahrsdoerfer classification.

CT Findings	Score
Stapes present	2
Middle ear space	1
Oval window open	1
Facial nerve normal	1
Malleus-incus complex present	1
Mastoid well-pneumatized	1
Incus-stapes connection	1
Round window normal	1
Appearance of external ear	1
